# Impact of a transformative health literacy model for Thai older adults with hypertension

**DOI:** 10.1186/s13104-024-06782-z

**Published:** 2024-09-27

**Authors:** Pilaiporn Sukcharoen, Nanchatsan Sakunpong, Jidapa Polruk, Sureeporn Chumdaeng

**Affiliations:** 1 Faculty of Nursing, Suratthani Rajabhat University, Suratthani, Thailand; 2 Behavioral Science Research Institute, Srinakharinwirot University, Bangkok, Thailand

**Keywords:** Health literacy, Transformative learning, Thai hypertension patients, Health and well-being

## Abstract

**Background:**

Hypertension is the important risk factor for cause disability and death, particularly if there is a loss of self-care knowledge. Health literacy encompasses the comprehension and awareness of health-related information, which is beneficial for managing the health of older adults with hypertension. Therefore, the objective of this study was to examine the impact of a transformative health literacy model to develop the health literacy levels among Thai senior citizen with hypertension.

**Method:**

This research employed an experiment. Thirty-six participants engaged in the transformative health literacy model. The instrument is the health literacy in hypertension scale, which had acceptable reliability and validity.

**Results:**

The study revealed that the level of health literacy in the post-test and follow-up phases of the experimental group who received the health literacy promotion model was significantly higher than the pre-test level of health literacy at a significance level of 0.05.

**Conclusion:**

The study outcomes create a new pathway to enhancements of health literacy in Thai older adults with hypertension.

## Introduction

Thai society is moving towards an aging society continuously due to the rapidly changing population structure. Thai people are living longer and mortality rates are decreasing due to efficient medical technology systems. This results in Thailand's future being filled with a predominant elderly population group, with a tendency to transform into a fully aged society and ultimately become a super-aged society [1, 2]. Furthermore, the physical decline and reduced bodily functions that come with old age often lead to the majority of elderly individuals suffering from diseases of the circulatory system, particularly hypertension, which may be linked to stroke and ultimately result in being bedridden and unable to care for themselves [2–4].

The medical system highlights the significant global impact of chronic diseases, with a particular emphasis on hypertension. This condition affects more than one billion individuals worldwide, including a notable burden of 10% in Surat Thani province, Thailand [5, 6]. Hypertension, if left untreated, can result in severe consequences such as heart disease, stroke, kidney damage, vision impairment, and an increased risk of aneurysm [7]. Hypertension-related fatalities contribute to 25.3% per 100,000 people, causing 9.4 million deaths, half from strokes [5, 7]. Especially among the elderly population, the prevalence of high blood pressure reaches up to 5 percent. If it cannot be controlled, it may lead to the occurrence of stroke and become a burden for the family [1, 5, 7]. Therefore, it is imperative to establish interventions aimed at both preventing the onset of disease and mitigating the impact of the disease on health to prevent it from becoming more severe [5–8].

Health literacy is vital in healthcare, encompassing cognitive abilities and skills. It involves acquiring, understanding, evaluating, and applying health information, enabling informed decisions [9–12]. Health literacy empowers patients to navigate medical complexities, access healthcare resources, and manage health actively, fostering empowerment [10]. This is particularly relevant for patients with chronic conditions, such as those afflicted with hypertension. Some studies have found that individuals with high blood pressure who possess good health literacy may have an impact on preventing the onset of disease or reducing the severity of the disease that has already occurred [11–14]. The health literacy skills can create the awareness of health, and improve self-care behaviors to maintain good health for hypertension patients [12, 13].

Transformative learning offers an alternative educational paradigm that can lead to cognitive and behavioral shifts. Grounded in self-awareness and critical introspection, this approach facilitates positive behavioral changes by challenging existing beliefs, promoting openness, and embracing new perspectives, contributing to the improvement of hypertension management [11–14]. Furthermore, the social cognitive learning theory asserts that individuals acquire knowledge and skills by engaging in observational learning, replicating seen behaviors, and receiving feedback on their own actions [15–17]. According to this idea, it is proposed that individuals have the potential to improve their health literacy through the process of seeing and emulating the health behaviors of individuals [16, 17]. Therefore, this research employed a learning process utilizing the transformative learning theory and social cognitive learning theory to enhance health literacy, aiming to facilitate a transformative change in the health literacy levels of individuals with high blood pressure. This is anticipated to contribute to preventing or mitigating the severity of the consequences associated with high blood pressure.

Based on previous research, it has been identified that there is lack of research support for the theoretical foundations of health literacy programs in individuals with high blood pressure [13, 18–22]. Consequently, this research aims to fill these knowledge gaps by developing a health literacy program based on the transformative learning model and social cognitive learning theory [15, 16, 23–30] to develop the health literacy among Thai hypertension older adult patients.

### Conceptual framework

**Fig.1:**
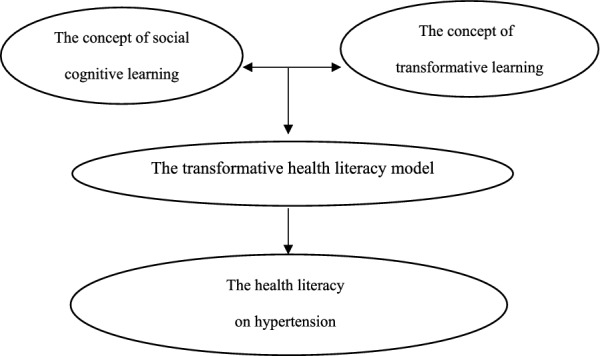
Conceptual Framework. The conceptual Framework of this research was the concept of social cognitive learning and the concept of transformative learning

The study's framework encompasses social cognitive learning theory [15, 16] and transformative learning concept [31–34]. The transformative learning model emphasizes the importance of critical thinking and reflection, allowing individuals to challenge their existing beliefs and perspectives. Also, social cognitive learning theory posits that people learn through observing others, imitating their behaviors, and receiving feedback on their own actions. This theory suggests that individuals can enhance health literacy by observing and modeling the health behaviors of others who are knowledgeable and skilled in managing health effectively [15, 16]. By incorporating social cognitive learning theory and transformative learning model into health literacy interventions, individuals may develop the necessary skills to make informed decisions about their health. Illustrated in Fig. 1.

## Methods

This study employed an expepriment. All procedures adhered to guidelines, and informed consent was obtained from participants and/or their guardians.

### Experiment and number of replicates

The research was conducted within the community of Surat Thani province. The number of replicates consisted of 36 Thai hypertensive older adult patients who were over 60 years old. Inclusion criteria include at least ten years of hypertension experience, good communication, being Thai hypertension older adult patients in the community, and having motivation to attend the study. Exclusion criteria include at request withdrawal or decline consent to participate in the research.

### Study tools

Research instruments were comprised of (1) the transformative health literacy model and (2) the health literacy in hypertension scale. In this research, the transformative health literacy model is an intervention provided to the sample group, and the health literacy scale is used to assess the effectiveness of this model.

### The transformative health literacy model

The transformative health literacy model emerged from an extensive review of literature encompassing transformative learning, and social cognitive learning theory [15, 16, 31–34]. This model focuses on Bandura's social cognitive theory, highlighting observational learning, self-efficacy beliefs, and the interplay of personal factors, environment, and behavior. The model underscores health-related knowledge acquisition's transformative nature, promoting informed choices for optimal health [15, 16, 31, 32].

The model followed a structured approach involving four steps, each spanning 4 h for a total of 16 h: (a) Teaching hypertension disease knowledge, (b) Communication and sharing experiences, (c) Analysis and discussion of hypertension information, (d) Decision-making for behavior changes.

### The health literacy in hypertension scale.

The health literacy in hypertension scale, utilized for data collection as a primary outcome, was developed from literature and research on health literacy and hypertension. It included 27 items measured on a 5-point Likert scale. Content validation involved three health literacy and two hypertension experts, and each item had the index of item-objective congruence (IOC) value higher than 0.05. A pilot test with 30 separated Thai hypertension patients yielded a Cronbach’s alpha value of 0.89 for reliability In this regard, the criteria for interpreting the health literacy in hypertension scale are divided into 5 levels, an average score of 5.00–4.21 is considered the highest level, 4.20–3.41 is considered the high level, 3.40–2.61 is considered the moderate level, 2.60–1.81 is considered the low level, and 1.80–1.00 is considered the lowest level.

### Data collection

Prior to the transformative health literacy model, the experimental group's "health literacy in hypertension disease" was measured as pretest scores. During the experimental phase, the experimental group underwent the model. Post-experiment, scores were collected. A follow-up measurement occurred 2 months later using the same instrument due to previous research evidence suggesting that the use of transformative learning contributes to cognitive changes in research participants lasting for 2 months [32, 33]

### Data analysis

This study employed t-test dependence statistical analysis using statistical software.

## Results

The pre-test mean scores of health literacy related to hypertension for the experimental group have a higher average level of health literacy than the mean scores of the post-test and follow-up phases at a significance level of 0.05, as shown in Table 1.

**Table 1:** Mean scores of health literacy

Health literacy related to hypertension score	Experimental group (n = 36)		95% Confidence Interval for Mean	
	M	SD	Lower Bound	Upper Bound
• Before the experiment	66.94	3.83	66.87	67.36
• After the experiment	72.13	5.34	71.54	72.97
• Follow-up	70.58	4.66	69.69	71.79

## Discussion

The study's results shed light on the transformative health literacy model's effects on the health literacy related to hypertension. Notably, their health literacy related to hypertension significantly improved at post and follow-up phase of experiment, reinforcing the model's efficacy. Developed by merging social cognitive learning theory and transformative learning, which aimed to foster learning, self-awareness, and behavioral changes [15–17, 31–34].

The transformative learning model, exemplified in this context, involved the evolution of perspectives through reflection and exposure to new experiences, such as video clips and shared experiences among group members facing similar challenges [25, 31–34]. The video clip covered information on hypertension, complications of hypertension, and how to manage oneself while suffering from hypertension. After playing the video clip, there was a group activity in which hypertension patients engaged in discussions and shared their feelings. This was done to utilize group processes to raise awareness among patients, encourage them to make changes in their health behaviors, and develop healthier habits. In particular, the acquisition of knowledge about hypertension by the group leader served as a basis for transformative experiences, facilitating the development of well-informed choices [31–34]. Therefore, this model contributed to hypertension patients gaining accurate knowledge about self-care methods, developing awareness of health adaptations, and fostering the need to improve health. This could be linked to the acquisition of self-care skills and the cultivation of improved health behaviors [20, 32].

Moreover, the effectiveness of Bandura's theory in the realm of health literacy is underscored. This theory emphasizes how observational learning, self-efficacy, and environmental factors collectively contribute to the acquisition of health knowledge and the decision-making process [15–17, 19, 25, 26]. This integrated perspective emphasizes the synergies between transformative learning, health literacy, and Bandura's theoretical framework, reinforcing their collective role in fostering informed health choices and outcomes.

In summary, the model can improve the health literacy related to hypertension among Thai hypertension older adult patients. Healthcare professionals can use this model, aiding in prevention and reducing the severity of hypertension-related issues in healthcare settings.

### Limitations

This study used data obtained only from Thai hypertension patients in Surat Thani province, in the southern region of Thailand. Additionally, the use of older adult participants may be another limitation in generalizing the research findings to the entire population of hypertensive patients.

## Conclusions

This study aimed to investigate the effects of a transformative health literacy model on Thai hypertension older adult patients. The results, indicated a significant improvement in health literacy related to hypertension among the experimental group. This research contributes to the existing knowledge base because it addresses a gap in health literacy programs for individuals with high blood pressure, offering theoretical foundations grounded in transformative learning and social cognitive learning theory.

## Data Availability

The datasets used and/or analyzed during the current study are available from the corresponding author upon reasonable request. All requests relating to data should be addressed to pilaiporn.navynurse@gmail.com.

## Appendix. Health literacy on hypertension scale

Please mark with a slash [ √] in the [] next to the statement that is most true about yourself, and please fill in the blank space.

**Table:**

Strongly Disagree	Disagree	Neutral	Agree	Strongly Agree
1	2	3	4	5

1. ผู้ที่มีความดันโลหิตสูงกว่า 140/90 mmHg ถือว่ามีความดันโลหิตสูง
2. โรคความดันโลหิตสูงทำให้เกิดหลอดเลือดอุดตัน, อัมพฤกษ์, อัมพาต
3. โรคความดันโลหิตสูงถ่ายทอดทางพันธุกรรม
4. ผู้ที่มีระดับความดันโลหิตสูงมีโอกาสเสี่ยงต่อการเกิดปัญหาของหัวใจและหลอดเลือด
5. ผู้ที่มีระดับความดันโลหิตสูงจะแสดงอาการผิดปกติทางร่างกายทุกราย
6. ผู้ที่ป่วยด้วยโรคความดันโลหิตสูงเสี่ยงต่อการเป็นโรคไต
7. ผู้ที่ป่วยด้วยโรคความดันโลหิตสูงไม่เสี่ยงต่อการเป็นโรคมะเร็ง
8. ผู้ที่ป่วยด้วยโรคความดันโลหิตสูงควรรับประทานยาต่อเนื่องทุกวัน
9. การหลีกเลี่ยงการรับประทานอาหารที่มีรสเค็มช่วยควบคุมความดันโลหิต
10. การออกกำลังกายอย่างสม่ำเสมอช่วยควบคุมความดันโลหิต
11. กรณีที่มีภาวะอ้วน การลดน้ำหนักช่วยควบคุมความดันโลหิต
12. การงดสูบบุหรี่ช่วยควบคุมความดันโลหิต
13. ฉันสามารถค้นหาข้อมูลเกี่ยวกับการดูแลสุขภาพเมื่อป่วยด้วยโรคความดันโลหิตสูง
14. ฉันพร้อมที่จะเปิดรับข้อมูลสุขภาพในโรคความดันโลหิตสูงเสมอ
15. ฉันไม่สามารถค้นหาแหล่งบริการสุขภาพที่จะให้การช่วยเหลือ หรือให้คำแนะนำแก่ฉัน
16. ฉันสามารถปฏิบัติตามคำแนะนำในเอกสารหรือแผ่นพับที่เกี่ยวข้องกับการดูแลสุขภาพ
17. ฉันเข้าใจในข้อมูลเกี่ยวกับโรคความดันโลหิตสูง ที่เผยแพร่ทางโทรทัศน์ วิทยุ สื่อออนไลน์
18. เมื่อมีข้อมูลใหม่ในโรคความดันโลหิตสูงเข้ามา ฉันจะตรวจสอบความถูกต้องของแหล่งที่มาของข้อมูล
19. ฉันจะไม่สอบถาม หรือขอคำปรึกษาจากผู้รู้หรือผู้ให้บริการสุขภาพเกี่ยวกับข้อควรปฏิบัติเมื่อป่วย
20. ฉันสามารถติดต่อสื่อสารกับบุคคลหรือกลุ่มคนที่มีความรู้ในโรคความดันโลหิตสูง
21. ถ้าฉันต้องการความช่วยเหลือ ฉันมีญาติหรือเพื่อนที่พร้อมจะช่วยเหลือฉันเสมอ
22. ฉันสามารถถ่ายทอดข้อมูลด้านสุขภาพเกี่ยวกับโรคความดันโลหิตสูงให้บุคคลอื่นยอมรับ
23. ฉันสามารถสนทนาแลกเปลี่ยนความรู้หรือแนวทางปฏิบัติเกี่ยวกับการดูแลตนเอง
24. ฉันไม่มีข้อมูลทางสุขภาพที่เกี่ยวข้องกับโรคความดันโลหิตสูง และไม่สามารถจัดการกับภาวะสุขภาพ
25. ฉันตั้งเป้าหมายที่จะออกกำลังกาย ดูแลสุขภาพ และจะทำให้ได้ตามที่ตั้งใจไว้
26. ขณะป่วยด้วยโรคความดันโลหิตสูง ฉันให้เวลากับการทำกิจกรรมเพื่อดูแลสุขภาพ
27. ขณะป่วยด้วยโรคความดันโลหิตสูง ฉันหมั่นสังเกตความผิดปกติของร่างกาย และจิตใจ
